# Emerging Connections of S1P-Metabolizing Enzymes with Host Defense and Immunity During Virus Infections

**DOI:** 10.3390/v11121097

**Published:** 2019-11-27

**Authors:** Jennifer J. Wolf, Caleb J. Studstill, Bumsuk Hahm

**Affiliations:** Departments of Surgery & Molecular Microbiology and Immunology, University of Missouri, Columbia, MO 65212, USA; jjwqn8@mail.missouri.edu (J.J.W.); cjs3vb@mail.missouri.edu (C.J.S.)

**Keywords:** sphingosine 1-phosphate metabolizing enzymes, virus-host interactions, host defense mechanisms, innate immunity, sphingosine kinase, sphingosine 1-phosphate lyase, viral pathogenesis

## Abstract

The sphingosine 1-phosphate (S1P) metabolic pathway is a dynamic regulator of multiple cellular and disease processes. Identification of the immune regulatory role of the sphingosine analog FTY720 led to the development of the first oral therapy for the treatment of an autoimmune disease, multiple sclerosis. Furthermore, inhibitors of sphingosine kinase (SphK), which mediate S1P synthesis, are being evaluated as a therapeutic option for the treatment of cancer. In conjunction with these captivating discoveries, S1P and S1P-metabolizing enzymes have been revealed to display vital functions during virus infections. For example, S1P lyase, which is known for metabolizing S1P, inhibits influenza virus replication by promoting antiviral type I interferon innate immune responses. In addition, both isoforms of sphingosine kinase have been shown to regulate the replication or pathogenicity of many viruses. Pro- or antiviral activities of S1P-metabolizing enzymes appear to be dependent on diverse virus–host interactions and viral pathogenesis. This review places an emphasis on summarizing the functions of S1P-metabolizing enzymes during virus infections and discusses the opportunities for designing pioneering antiviral drugs by targeting these host enzymes.

## 1. Introduction

**Figure 1:**
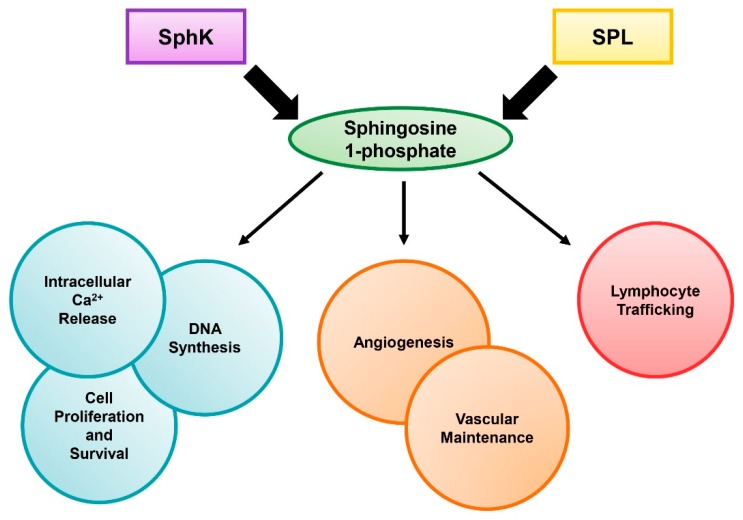
**The role of sphingosine 1-phosphate (S1P).** S1P levels are regulated by sphingosine kinase (SphK) and S1P lyase (SPL). The diverse functions of S1P on cell survival and host health are shown.

Sphingolipids are a class of lipids that have several significant roles in regulating cellular functions. The sphingolipid family is made up of diverse members, including sphingomyelin, ceramide, ceramide 1-phosphate, sphingosine, and sphingosine 1-phosphate (S1P). S1P is known to be an important signaling molecule that modulates multiple mammalian cellular processes, such as proliferation, survival, and migration, through both extracellular receptor-mediated and intracellular mechanisms (Figure 1) [1]. S1P has been shown to regulate the release of free calcium ions from the endoplasmic reticulum, which has extensive effects on cell proliferation [2,3]. Furthermore, S1P binding the S1P receptors (S1PR1-5) on the outer membrane of the cell signals through the Ras/Raf/extracellular-signal-regulated kinase (ERK) pathway, which stimulates DNA synthesis and increases cell growth, promoting cell survival [4,5]. This signaling increases S1P generation by sphingosine kinase (SphK), which in effect creates a positive feedback loop, and blocks the pro-apoptotic functions of ceramide [5,6]. In addition, S1P signaling through S1PR1 was shown to promote cell motility in a Rac-dependent manner [7]. S1P’s functions can also impact the organism as a whole. In several studies, S1P signaling was shown to be crucial for angiogenesis and vascular development, and lack of S1P caused increased vascular leakage [8,9,10]. Finally, S1P has been shown to play a large part in lymphocyte trafficking via the S1P gradient, which has been studied extensively in the functioning of sphingosine analog FTY720 [11,12]. Interestingly, both SphK and S1P lyase (SPL) have been shown to play important roles in the maintenance of the S1P gradient, which places these enzymes in a critical role during the host inflammatory response [13].

Because S1P has crucial functions in multiple cellular pathways, it has been a large target for curing human diseases. The first oral therapy with the sphingosine analog FTY720 is currently used in the clinic for the treatment of multiple sclerosis [14,15,16]. While FTY720 is known to functionally antagonize the S1P receptor to block T cell trafficking and help attenuate autoimmunity, the resultant immune suppressive condition was shown to cause increased susceptibility to viral diseases [17,18,19]. Currently, it is unknown whether the regulation of S1P metabolism, such as targeting of S1P-metabolizing enzymes, could relieve the side effects of FTY720-mediated increased viral diseases. Furthermore, SphK was shown to be overexpressed in several cancers [20,21,22,23,24]. Accordingly, SphK inhibitors have been evaluated for the treatment of cancers in clinical trials [22,25,26,27]. As patients could be persistently infected with viruses or exposed to viruses at any moment, it is imperative to understand how S1P-metabolizing enzymes affect virus pathogenesis and host defense to infections. Importantly, a growing body of studies indicate that many viruses regulate the enzymes in a positive or negative fashion to enhance virus pathogenicity, suggesting that these enzymes play a crucial role during virus infections. Thus, manipulation of S1P-metabolizing enzymes by itself has great potential for the development of new therapeutics against several viral diseases.

## 2. S1P-Metabolizing Enzymes During Virus Infections

**Figure 2:**
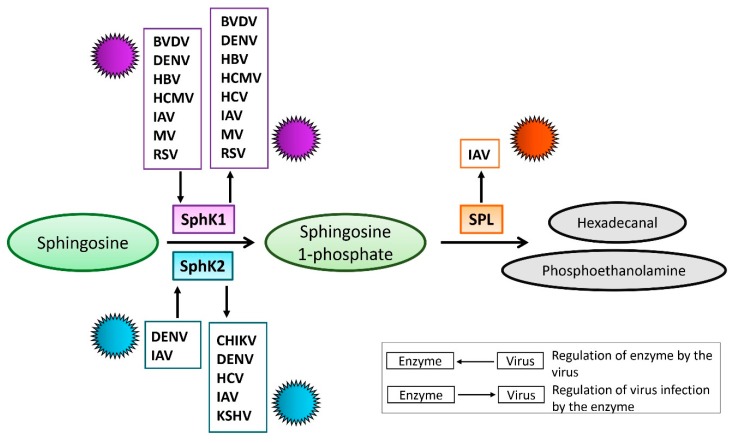
**Schematic diagram for the interaction between viruses and S1P-metabolizing enzymes.** The enzymatic function of sphingosine kinase (SphK) 1 and 2 is to catalyze the phosphorylation of sphingosine to form sphingosine 1-phosphate (S1P), while S1P lyase (SPL) irreversibly catalyzes the cleavage of S1P into hexadecanal and phosphoethanolamine. Depicted are viruses that were documented to either be affected by or regulate SphK1, SphK2, or SPL.

Sphingolipid levels are tightly regulated by sphingolipid-metabolizing enzymes, which are critical for maintaining cell homeostasis. Thus, S1P anabolism from sphingosine is mediated by SphK, and sphingosine 1-phosphate lyase (SPL) mediates S1P catabolism into phosphoethanolamine and hexadecanal [28,29] (Figure 2). There are two different SphK isoforms in humans, sphingosine kinase 1 (SphK1) and sphingosine kinase 2 (SphK2). The proposed cellular functions of SphK1 and SphK2 have similarities and distinct differences [29,30]. Intriguingly, the SphK isoforms and SPL have all been found to regulate virus propagation [31,32,33]. Furthermore, the levels and activation of SphK have been shown to be affected by several different viruses [34,35,36,37,38]. Therefore, manipulation of S1P-metabolizing enzymes may represent a virus-induced mechanism for promoting replication. This review focuses on summarizing the functions of SphK and SPL during viral infections and discusses opportunities for designing new potential therapeutics targeting these enzymes.

### 2.1. SphK During Virus Infection

SphK is essential for the production of intracellular and extracellular S1P. Both isoforms of SphK, SphK1 and SphK2, catalyze the phosphorylation of sphingosine to form sphingosine 1-phosphate (S1P) [39,40]. Despite conserved domains and similar enzymatic activity, these isoforms have distinct differences. SphK1 is known to promote cell survival and proliferation, but SphK2 has been shown to have both pro- and anti-apoptotic properties [41,42,43,44]. These two isoforms are also located in distinct subcellular compartments. SphK1 is in the cytosol and plasma membrane. SphK2 localizes primarily in the nucleus but can also be found in the cytosol and endoplasmic reticulum under certain conditions [42,45,46]. SphK1 has been shown to be critical for tumor necrosis factor-alpha (TNF-α) and nuclear factor kappa-light-chain-enhancer of activated B cells (NF-κB) signaling during inflammatory responses while both pro- and anti-inflammatory functions of SphK2 have been reported [41,47,48]. SphK2, but not SphK1, mediates the phosphorylation of FTY720 [49,50,51], an analogue of sphingosine and an immune modulatory drug used clinically for the treatment of multiple sclerosis [14,15,16]. Since the S1P biosynthesis pathway is crucial to the control of cell survival and apoptosis as well as other functions, both isoforms of SphK could be crucial targets for virus replication or inhibition (Figure 2). We discuss the interactions of SphK1 and SphK2 with different viruses in separate sections below.

#### 2.1.1. SphK1 in Virus Replication

Several studies have been performed regarding the role of SphK1 in the replication of certain viruses. SphK1 is tightly controlled by the cell, largely because of the several important functions it controls through S1P production [52], making it a prime target for viral manipulation.

Respiratory syncytial virus (RSV) infection in adenocarcinomic human alveolar basal epithelial (A549) cells was found to stimulate SphK1 activity as well as neutral ceramidase activity. This combination of effects decreased pro-apoptotic ceramide and enhanced anti-apoptotic S1P. Furthermore, the reduction of ceramide and increase of S1P were found to lead to the activation of pro-survival pathways, protein kinase B (Akt) and extracellular signal-related kinases (ERK), which enabled RSV-infected cells to remain viable during the infection. Chemical inhibition of SphK1 in RSV-infected A549 cells reduced cell survival, which suggests that increased SphK1 activity is important to promote the survival of RSV-infected cells. This study highlighted the importance of sphingolipid metabolites in cell survival mechanisms during RSV infection [38]. However, it remains to be studied how SphK1 activation and the resultant prolonged cell survival affect RSV propagation and viral pathogenicity.

Increased mRNA levels of hepatitis B virus (HBV) nonstructural protein, HBx, were positively correlated with an increase in SphK1 mRNA levels in clinical hepatocellular carcinoma patient tissues as well as HBx-transgenic mice [53]. A correlation was also discovered at the protein level utilizing these mice. HBx-transgenic mice were found to have reduced tumor growth when HBx was silenced. Transfection with increasing amounts of HBx-encoding plasmid resulted in increased SphK1 expression and promoter activity in vitro, but the silencing of HBx using small interfering RNA (siRNA) led to suppressed SphK1 expression and promoter activity. The authors recapitulated these findings in an HBx-overexpressing human liver cancer(HepG2) cell line. Transcription factor activating enhancer binding protein 2 alpha (AP2α) was found to directly interact with the SphK1 promoter in HBx-overexpressing HepG2 cells, and SphK1 promoter activity and SphK1 expression was suppressed when AP2α was silenced. The authors also observed that silencing SphK1 using siRNA inhibited the growth of hepatoma mediated by HBx in vitro and in vivo. These findings led the authors to conclude that HBx upregulates SphK1 through transcription factor AP2α, which promotes the growth of human hepatoma cells. Further study is required to determine the effects of SphK1 expression and activity on HBV replication and evaluate the viability of SphK1 inhibition as a potential therapeutic for hepatocellular carcinoma or chronic HBV infection.

Knockdown of SphK1 using siRNA was found to inhibit replication of two luciferase reporter hepatitis C viruses (HCV), genotype 1a virus H77S.3/GLuc and genotype 1b virus N.2/GLuc, in Huh-7.5 cells [54]. However, SphK1 knockdown increased replication of the HCV luciferase reporter virus HJ3-5/GLuc, which is an inter-genotypic chimera that contains the replicase of a genotype 2a HCV, and the cell culture-adapted genotype 2a JFH1 (JFH-QL/GLuc) virus. Differences in the HCV genotype likely contributed to the varying effects of SphK1 knockdown on HCV replication. However, it was unclear if the effects of SphK1 knockdown on cell culture-adapted HCV strains were due to SphK1 itself or the potential effects of SphK1 knockdown on S1P metabolism. A greater depth of research in this area could reveal this.

Late-stage bovine viral diarrhea virus (BVDV) infection induced a decrease of SphK1 activity, which was crucial for efficient BVDV replication [37]. Although SphK1 mRNA levels were not altered, a yeast two-hybrid screen showed that BVDV non-structural protein 3 can bind to and inhibit SphK1 enzymatic activity. When catalytically inactive SphK1 was overexpressed or when SphK1 was inhibited by using siRNA or a chemical inhibitor, BVDV replication was enhanced. Overexpression of SphK1 reduced the induction of apoptosis in cells infected with cytopathogenic BVDV, suggesting that that BVDV requires reduced SphK1 activity for virus-induced cell death.

Late-stage Dengue virus type 2 (DENV-2) infection in vitro was also found to lead to reduced SphK1 activity and TNF-α-induced enhanced apoptosis [35]. The authors observed that SphK1 mRNA levels remained unchanged during DENV-2 infection in vitro, similar to BVDV. Chemical inhibition of SphK1 after established DENV-2-infection resulted in enhanced cell death with no changes in infectious DENV-2 production [55]. SphK1 activity was not affected by DENV-2 NS3 protein but was instead reduced by the expression of 396 bases of the 3′UTR of DENV-2 RNA. DENV-2 RNA was found to colocalize and coprecipitate with eEF1A, which may activate SphK1. The authors proposed that the reduced SphK1 activity late in DENV-2 infection was a result of DENV-2 out-competing SphK1 for eEF1A binding in order to use eEF1A for its own replication. However, the same research group reported that during the early stages of DENV infection of endothelial cells, SphK1 activity was increased [56]. The authors noted that this was associated with enhanced TNF-α-induced permeability of the endothelial cells during early infection, decreasing the barrier integrity of the cells, but the barrier integrity was normalized by late-stage infection. Thus, the authors surmised that while the integrity of the endothelial barrier is compromised during early stage DENV infection as the result of an acute response, the increased SphK1 activity could restore normal vascular barrier function. The same group later found that overexpression of SphK1 does not alter DENV infection, but chemical inhibition of SphK1 resulted in reduced DENV RNA and infectious virus production [57]. However, they observed that DENV replication was increased in primary murine embryonic fibroblasts deficient in SphK1, which seemed to be due to a decreased basal level of type I interferon (IFN) receptor and IRF1 through genetic modification and adaptation. It remains unclear how complete deletion of SphK1 in murine fibroblasts affected the host innate immune system. Collectively, these studies suggest that SphK1 could have multiple functions during DENV infection, presumably depending on the stage of infection and cell type.

In contrast to BVDV, our lab found that measles virus (MV) increased levels of SphK1 and pSphK1 in B lymphoblastoid B95-8 cells, and SphK inhibition was shown to impair MV replication in human bronchioalveolar H358 cells as well as B95-8 cells [58]. Furthermore, the inhibition of MV replication was associated with suppressed NF-kB activation. In another study, SphK activation was shown to enhance the velocity of MV-infected dendritic cells (DCs) [59]. The authors utilized a 3D respiratory tract model to demonstrate that MV-infected DCs migrate to epithelial cells to transmit MV. These results suggest that SphK plays a key role in MV pathogenesis by contributing to the migration of MV-infected DCs, resulting in efficient viral spread. Therefore, MV appears to have multiple means to use SphK for its own benefit.

Our lab has also found that inhibition of SphK1 reduced the synthesis of influenza A virus (IAV) RNAs and proteins as well as suppressed IAV-induced NF-κB activation [34]. Treatment with an SphK1 inhibitor suppressed nuclear export of the viral ribonucleoprotein complex and interfered with the activation of Ran-binding protein 3 (RanBP3), which is a cofactor of chromosome region maintenance 1 (CRM1). Inhibition of SphK1 was found to inhibit the CRM1-mediated nuclear export of the IAV ribonucleoprotein complex. SphK1 inhibition also altered the phosphorylation of ERK, p90RSK, and AKT, the upstream signals of RanBP3/CRM1 activation. In a later study, our lab found that inhibition of SphK1 protects mice against lethal challenge with IAV [60]. Therefore, harnessing SphK1 appears to be critical for effective IAV infection in vitro and in vivo. Our lab has previously detailed how SphK1 can affect influenza virus replication by regulating cellular signaling pathways, such as the NF-κB pathway, in a previous review [61].

#### 2.1.2. SphK/S1PR in Virus Infection

Regulation of SphK has been shown to affect S1P levels, which regulates S1PR-mediated signaling or intracellular functions of S1P/SphK. Several studies determined the direct involvement of the S1PR-mediated regulation of virus infections, which are discussed in this section.

Human cytomegalovirus (HCMV) infection increased SphK1 enzymatic activity and the resultant S1P levels, and the inhibition or downregulation of SphK1 interfered with HCMV replication [36]. In addition, neutralization of extracellular S1P using an anti-S1P antibody or an inhibitor of S1P receptor 2 (S1PR2) decreased HCMV replication, indicating that S1P receptor 2 signaling is crucial for efficient HCMV replication [62]. Therefore, the SphK or S1PR2 signaling pathways could be targeted for the design of new therapeutic interventions against HCMV infection.

While SphK is upregulated by influenza viruses, ligation of S1P receptor 1 (S1PR1) was also shown to influence influenza virus pathogenesis by regulating host inflammatory responses [63,64,65]. A discussion of S1PR1 and its effects on IAV can be found in a book titled *Sphingosine-1-Phosphate Signaling in Immunology and Infectious Diseases* authored by Dr. Oldstone and Dr. Rosen [66].

In the case of human immunodeficiency virus (HIV) infection, S1PR1 was reported to dimerize with CCR5 on primary CD4 T cells; although, this does not seem to affect the entry of HIV into cells [67]. When human osteosarcoma or MT4 cells were engineered to express S1PR1, they were more permissive to HIV infection, which appeared to be dependent on the increased activation of NF-κB signaling. Furthermore, FTY720 impaired HIV-1 replication in monocyte-derived dendritic cells and inhibited HIV infection of humanized mice, which are severe combined immunodeficiency mice engrafted with human peripheral blood mononuclear cells. These results suggest that S1P receptor signaling is important for productive infection of HIV. It has also been shown that in untreated viremic HIV-1 patient lymph nodes, CD4 naïve and central memory T cells as well as CD8 central memory T-cells had significantly diminished Akt phosphorylation responses following exposure to S1P [68]. This suggests that uncontrolled HIV infection could lead to T cells that are unable to efficiently migrate out of inflammatory lymph nodes. However, FTY720 did not seem to have any therapeutic effects on simian human immunodeficiency virus (SHIV) infection of rhesus macaques [69,70]. The possible role of S1P-metabolizing enzymes in HIV or SHIV infection has yet to be reported.

#### 2.1.3. SphK2-Virus Interaction

The second isoform of sphingosine kinase, SphK2, shares conserved SphK domains with, and can perform a similar enzymatic reaction, to SphK1. Differences arise in SphK2′s localization as well as preference for D-erythro-dihydrosphingosine and ability to phosphorylate d,l-threo-dihydrosphingsoine [71]. Since SphK2 has been shown to play a unique role in cells, it may represent a crucial target for viral pathogens as well as therapeutics in the fight for/against viral replication.

A recent study in our lab has indicated that SphK2 is beneficial for IAV replication in cells and in mice [60]. The levels of SphK2 and phosphorylated SphK2 were significantly increased in A549 cells upon infection with IAV H1N1 as well as IAV H3N2 and influenza B virus. While overexpression of SphK2 increased IAV replication, use of an SphK2-specific inhibitor, ABC294640 (ABC), or an siRNA against SphK2 resulted in decreased viral replication. Importantly, treatment of IAV-infected mice with ABC led to decreased virus titers and increased mouse survival. This study has a significant effect in that it identifies a new host target for controlling influenza virus infection, which may provide a novel therapeutic to complement current strategies. However, it is yet to be determined how IAV utilizes host SphK2 to increase viral replication. Since SphK2 can control gene expression and cell cycling in the nucleus of cells [72], it is possible that IAV components directly or indirectly interact with SphK2 and cause changes in SphK2′s activation or localization in an effort to push the host cell to a more beneficial state for viral replication. Alternatively, it would be interesting to determine if SphK2 can directly regulate replication of the viral genome, which is an idea seen with other viruses discussed below. Overall, this study provides a system for understanding what effect IAV has on the regulation of the host sphingolipid biosynthesis pathway as well as giving a platform for further analysis into the pathways SphK2 may be involved with in cells.

SphK2 has also been shown to play a positive role during chikungunya virus (CHIKV) infection. In a study by Reid and colleagues, utilization of an siRNA against SphK2 decreased the viral RNA copy number of CHIKV in HeLa cells, and ABC treatment led to a decrease of CHIKV infection in HepG2 cells [73]. However, SphK1 knockdown did not affect viral replication, suggesting a specific role of SphK2 during CHIKV infection. Affinity purification-mass spectrometric analysis revealed an enriched association of SphK2 with host mRNA processing and gene expression factors during CHIKV infection. Interestingly, SphK2 was shown to localize to distinct punctate structures within the cytoplasm of infected cells. In these structures, SphK2 localized with CHIKV dsRNA during early infection stages, leading the authors to suggest a potential role for SphK2 in regulating viral gene expression. Finally, SphK2 was shown to be recruited to CHIKV viral replication complexes at the plasma membrane, potentially by CHIKV nonstructural protein 3, where it also co-localizes with G3BP, a helicase. Future work will be needed to fully determine SphK2′s role in promoting CHIKV replication by co-localizing with the CHIKV replication complex during infection.

In similarity to SphK1, the interaction of SphK2 with DENV has been investigated. In vitro studies utilizing an RNAi screen for apoptosis-related genes during DENV infection of human liver cancer (Huh7) cells revealed that SphK2 may play a role in caspase 3-mediated apoptosis [74]. This effect was seen with all four DENV serotypes. Specific knockdown of SphK2 or treatment with ABC led to a reduction in caspase 9 activity and apoptosis of DENV-infected cells. The authors also noted that SphK2 appeared to increase in the cytoplasm of DENV-infected liver cells, with possible co-localization with DENV E protein. This could point to a potential mechanism for SphK2′s regulation of apoptosis in these cells. However, SphK2 knockdown or inhibition by ABC did not affect DENV replication or protein levels in multiple cell types [75]. Furthermore, SphK2-deficient mice did not display differences in survival or DENV RNA levels in their brains compared to wild-type mice. Based on these studies, it does not appear that SphK2 plays a role in the direct replication process of DENV. However, the initial study does indicate a potential mechanism whereby DENV proteins interact with host SphK2, leading to increased apoptosis. It would be beneficial to look further into this host–virus interaction to determine a potential mechanism for DENV-induced regulation of host liver cell apoptosis via SphK2. Furthermore, SphK2 inhibition represents a potential therapeutic for DENV-associated pathology in the liver.

Interestingly, SphK2 has been shown to function differently in response to different genotypes of HCV. In one study, use of the SphK inhibitor, SKI, which the authors stated has preferential inhibitory functions against SphK2 at the concentrations used, led to an increase in the replication of the cell-culture adapted genotype 1 HCVs in Huh-7.5 cells [54]. However, a chimeric genotype 2 virus, HJ3-5/GLuc, was not suppressed by SKI treatment. These results were assessed by measurement of the luciferase reporter activity that reflects viral replication as well as analysis of viral protein levels. Targeted knockdown of SphK2 had a similar regulatory effect on HCV replication to the inhibitor treatment. These findings were confirmed with wild-type HCVs and in primary human hepatocytes. The authors of this study implicated SphK2′s regulation of lipid peroxidation, which has previously been shown to negatively affect the replication of genotype 1b HCV [76,77]. The direct function for SphK2 in the mechanism described in this study has yet to be determined. Nevertheless, the potential for SphK2′s regulation of host cellular lipid peroxidation, which can regulate viral replication, is a novel concept and warrants further investigation.

Since SphK2 has been implicated in the modulation of several forms of cancer, it is not altogether surprising that SphK2 has been shown to play a role in latency for the oncogenic virus Kaposi’s sarcoma-associated herpesvirus (KSHV). In a group of studies, treatment of KSHV-infected patient-derived primary effusion lymphoma (PEL) cell lines with ABC led to increased apoptosis in in vitro and in murine-graft in vivo experiments [78]. In subsequent studies utilizing primary human endothelial cell lines, SphK2 played a positive role in NF-κB activation, which is known to promote KSHV latency [79,80,81]. One of the most interesting aspects of this study was the finding that SphK2 increases the levels of certain viral miRNAs that target KSHV lytic genes. Turning off these genes prevents the initiation of host cell lysis and allows for viral latency. Thus, inhibition of SphK2 increased the death of KSHV-infected cells. While the data imply that the production of S1P by SphK2 is crucial for regulation of viral RNAs, a direct mechanism by which SphK2 can regulate viral gene expression is not yet known. Furthermore, treatment of PEL cells with ABC resulted in an increase in ceramide and ceramide synthase species [78,82]. Increasing levels of these ceramide species were also shown to promote apoptosis in KSHV-infected cells via regulation of the KSHV lytic genes, which implies a deeper connection between KSHV infection and the sphingolipid metabolism pathway. However, a more recent study utilizing the TIVE-LTC cell lines, which are useful for studying KSHV latency, indicated that ABC-induced death of KSHV-infected cells occurred via autophagy rather than apoptosis [83]. Thus, KSHV-induced cell death mechanisms and the role of SphK2 in these processes remain to be further examined. Taken together, these studies show an interesting mechanism by which KSHV may promote viral latency via regulation of host SphK2 and provide a potential therapeutic target for alleviating KSHV-associated tumors.

### 2.2. S1P Lyase During Virus Infection

SPL is known for regulating cell migration, differentiation, survival, and complex physiological processes [84]. In the sphingolipid biosynthesis pathway, SPL irreversibly catalyzes the cleavage of S1P to hexadecenal and phosphoethanolamine. Based on studies performed with cancer and IAV, which are outlined below, SPL shows great research potential for virus–host interactions.

Molecular genetic studies performed in the model organism *Dictyostelium discoideum* discovered that regulation of SPL or SphK leads to altered cellular sensitivity to cisplatin, a chemotherapy drug [85]. The finding was recapitulated in human cell lines. For example, SPL-overexpressing human embryonic kidney (HEK) cells, which were generated by Dr. Alexander’s group, were shown to be more sensitive to anti-cancer drugs compared to control cells [43]. This study triggered a follow-up investigation into the possible role of SPL in oncogenesis and cell survival. Thus, SPL was found to be downregulated in colon cancer and prevent colon carcinogenesis [86]. SPL has also been shown to have interesting immunologic functions, including the regulation of mature lymphocytes exiting the thymus. Moreover, genetic mutations of SPL were found in humans, linking SPL dysfunction to human diseases, including nephrotic syndrome, immunodeficiency, congenital brain malformation, and primary adrenal insufficiency [87,88,89,90]. The biological functions of SPL in human health have been well-reviewed by Dr. Saba’s group [84,91,92,93,94,95].

Unlike the previously described increased sensitivity of SPL-overexpressing cells to the anticancer drug cisplatin, SPL-overexpressing HEK cells were more resistant to cell death when they were infected by influenza A virus [31]. This changed susceptibility to cell death seemed to be due to overexpressed SPL regulating IAV replication. The antiviral activity of SPL was supported by transient overexpression of SPL in human lung epithelial cells. Furthermore, when cells were engineered to be SPL deficient by utilizing CRISPR/Cas9 genome editing technology, IAV replication and viral propagation substantially increased, confirming the antiviral function of SPL.

Importantly, the underlying mechanism of this resistance [33] indicates that SPL is a host-protective regulator that enhances the production of type I interferon (IFN) upon IAV infection or cellular recognition of influenza viral RNAs. This mechanism occurs through the interaction of SPL with inhibitor of nuclear factor kappa-B kinase subunit epsilon (IKKε), a kinase important for IFN production. While two kinases, TANK-binding kinase 1 (TBK1) and IKKε, are known to be crucial for the production of type I IFNs, SPL was shown to interact with IKKε but not with TBK1. In support of this observation, SPL increased IKKε activation but not TBK1 activation. Many of the observed effects of SPL modulation can be attributed to the alteration of intracellular S1P levels. However, the pro-IFN function of SPL was independent of the ability of SPL to catalyze S1P cleavage and appeared to be mediated via interaction with IKKε. IKKε was also shown to be critical for SPL’s anti-influenza activity in both HEK cells and lung A549 cells. It would be interesting to further characterize and refine the interaction between IKKε and SPL by using structural methods, such as cryogenic transmission electron microscopy or crystallography, in order to more completely understand the structural basis behind it.

IKKε gene knockout (KO) mice were reported to be more susceptible to influenza virus infection [96], which demonstrates the antiviral activity of IKKε. It would be interesting to investigate the role of SPL during influenza infection in vivo in order to further define the interaction between IKKε and SPL. Although both IKKε and TBK1 can activate IRF3, a transcription factor crucial for IFN production, the discrete regulatory mechanism of these two kinases remains poorly understood. Further exploration of the specific regulation of the IKKε-dependent, but TBK-independent, IFN response by SPL could increase our knowledge about the IFN production pathway.

The function of SPL has not been widely studied in the context of viral infection, presumably due to the lack of research resources and tools, such as an SPL-specific inhibitor that can be utilized in vitro. Furthermore, SPL KO mice die when they reach 2 to 4 weeks of age [93], which makes it difficult to use them for studying viral pathogenesis. However, SPL gene (Sgpl1) floxed mice have been created [86,97], which will be useful for investigating the role of SPL in antiviral defense and host innate immune signaling in the future.

## 3. Perspectives

S1P-metabolizing enzymes appear to impact the pathogenicity of many viruses by regulating the virus replication processes and/or controlling host innate defense to virus infections. Additionally, the functions of S1P–S1P receptor interaction in host immunity, such as T cell trafficking and programming of cytokine repertoire, may affect virus pathogenesis. Since S1P-metabolizing enzymes have a crucial role in diverse cellular processes, many viruses regulate these enzymes to benefit their own propagation. In contrast, the role of S1P lyase in enhancing the antiviral type I IFN response can affect cellular defense against virus propagation, which remains to be further studied. Investigating the functions of these enzymes in virus infections could help us better understand viral pathogenesis and host responses during viral infections. Exploring the intricate interaction between viruses and S1P-metabolizing enzymes could also lead to the design of new therapeutics against some viral diseases.

While many studies have been conducted using in vitro cell culture experimentation, several lines of investigation demonstrated the importance of S1P and its metabolizing enzymes during virus infections in humans or in vivo mouse models. For instance, SphK1 levels were shown to be increased in clinical hepatocellular carcinoma patient tissues as well as HBx-transgenic mice [53]. Modulation of SphK1/2 or S1P1 could confer protection to influenza virus-infected mice [60,66]. Furthermore, during FTY720 (fingolimod) therapy, patients with multiple sclerosis become susceptible to viral disease, possibly because of the immune suppressive properties of the treatment [17,18,19]. Recently, FTY720 treatment was shown to increase the incidence of chronic warts, which is associated with human papilloma virus (HPV) infection [98]. As HPV infection causes cervical cancers and anogenital cancers, it would be very beneficial to understand how S1P metabolism regulates HPV infection. These studies are important because they highlight the potential for targeting S1P-metabolizing enzymes as therapeutics during human viral infections.

Overall, the therapeutic potential of inhibitors that block the activity of S1P-metabolizing enzymes and S1PR for the treatment of viral diseases and immunologic disorders necessitates a deeper understanding of viral interaction with the sphingolipid network. Developing and utilizing diverse research tools and animal models, including conditional knockout mice lacking SphK or SPL, could allow us to comprehensively understand the role of these enzymes during virus infections.
